# A Method of Preparing Dental Tissues for Microscopical Examinations

**Published:** 1887-01

**Authors:** W. C. Brittan


### A Method of Preparing Dental Tissues for Microscopical Examination.

BY W. C. BRITTAN.

To the histologist there are no tissues of the animal body more instructive, or to the appreciative microscopist more beautiful, than those of the dental organs. Having, as I believe, been more than ordinarily successful in the preparation of these tissues for microscopical examination, I give the method which, although somewhat tedious, will be found, I think, to well repay for the time spent in the preparation. I have some slides prepared by this method, which are to me more valuable than any work of art by old masters or modern ; valuable because they reveal nature and her secluded and mysterious methods in the production of these organs in as perfect a manner as possibly could be hoped for.

First, however, I would acknowledge my indebtedness to Mr. E. H. Sargent, of Cornell University, whose work in this direction is most truly of a superior order. Although the method here given differs materially from that practiced by him, still it is to him I am indebted for the knowledge that such work was possible.

The qualifications for this kind of work are, patience, perseverence, and an inherent love for it, if you have these go ahead, if not, don't try. With some small animal for your subject, as a dog, cat, or rat—cats are always handy and make good subjects—preferably your neighbors—chloroform, and inject from the heart. Beals' injection medium is good. When thoroughly congealed, dissect out such parts as you wish to use, remove all adherent flesh and place in Muler's fluid for two weeks, then transfer to alcohol ninety-five per cent. for about two weeks more. You may now, with a fine sharp file, cut down the bone, jaw, and teeth, until, by holding to the light, the pulps are visible, being very careful to keep them constantly wet with alcohol while this is being done. Wash them thoroughly and place in oil of cloves until thoroughly transparent, which will take two or three days, then transfer to a very thin solution of balsam in benzole, after a few days pour off a part and add more balsam, gradually getting them into pure balsam. The object being to effect a thorough permeation of the balsam through the whole piece. This accomplished, place the piece in some shallow dish, with balsam enough to cover it, and by sand or water bath, evaporate to hardness, being careful not to raise the heat above 110° F. You may now proceed to make the section which is done by filing, cutting, and dissecting, using much care and frequent examination, for the different parts which you may wish to preserve do not always lie in the same plane, consequently do not try to make them very thin, when they show what you wish to see be satisfied, when the section suits you dissolve out the balsam with benzole, place again in oil of cloves for a few hours and mount, using as little heat as possible. As these sections are always unavoidably somewhat thick, I mount in a cell ground into the slide, which allows the cover-glass to be brought down close and makes a handsome mount when finished. Now, all this as before stated, is somewhat tedious and takes time, besides a great amount of patience, but the results most amply pay for all the trouble.

As an example of what may be done, I have a section of the jaw of a young animal containing two deciduous molars just erupted and about half developed, also the germs of the permenant molars, which are to replace them, beautifully showing the folicles, enamel organs and pulps with their points of dentification etc., with their vessels all beautifully injected. This slide alone I should hold cheap, though it should take a year's time for its preparation.

I find that sections of these tissues prepared in this way afford facilities for study far superior to any other methods known to me.

